# Triple-negative and HER2-positive breast cancers found by mammography screening show excellent prognosis

**DOI:** 10.1007/s10549-020-06060-z

**Published:** 2021-01-08

**Authors:** Johanna Alanko, Minna Tanner, Ritva Vanninen, Anssi Auvinen, Jorma Isola

**Affiliations:** 1 Laboratory of Cancer Biology, Faculty of Medicine and Health Technology, Screening Clinic of Terveystalo, Tampere University, Tampere, Finland; 2 Department of Oncology, Faculty of Medicine and Health Technology, Tampere University and Tays Cancer Centre, Tampere, Finland; 3 Department of Clinical Radiology, School of Medicine, University of Eastern Finland, Kuopio University Hospital, Kuopio, Finland; 4 Faculty of Social Sciences (Health Sciences), Tampere University, Tampere, Finland; 5 Laboratory of Cancer Biology, Faculty of Medicine and Health Technology, Jilab Inc., Tampere University, Tampere, Finland

**Keywords:** Mammography screening, HER2 positive, Triple-negative breast cancer, Prognosis

## Abstract

**Purpose:**

Our purpose was to explore the prognosis of aggressive breast cancers of the HER2 oncogene amplification (HER2 +) and triple-negative (TN) subtypes detected by screening, as well as the prognosis of interval cancers (clinically due to symptoms between screening rounds) and cancers in screening nonparticipants.

**Methods:**

The study population comprised of 823 breast cancers in women aged 50–69 years from 2006–2014. Of these, 572 were found by screening mammography (69%), 170 were diagnosed between the screening rounds (21%), and 81 were diagnosed in women who did not participate in the screening program (10%).

**Results:**

The majority of all HER2 + (59%) and TN cancers (57%) in this age group were detected by screening. Screen-detected HER2 + tumors were small (median 12 mm), and node-negative (84%). During a median follow-up of eight years, the distant disease-free survival of screen-detected HER2 + and TN cancers was better than that of interval and nonparticipant cancers (age-adjusted HR = 0.16, 95% CI 0.03–0.81 and HR = 0.09, 95% CI 0.01–0.79, respectively). In nonparticipants, the distant disease-free survival of these cancers was worse than in participants (age-adjusted HR = 2.52, 95% CI 0.63–10.11 and HR = 5.30, 95% 1.16–24.29, respectively).

**Conclusion:**

In the 50–69 age group, the majority of HER2 + and TN cancers can be found by a quality assured population-based mammography screening. Despite their generally aggressive behavior, after a median follow-up of 8 years, distant disease-free survival was over 90% of these cancers detected by screening. The worst prognosis of these cancers was in women who did not participate in screening.

## Background

Breast cancer screening aims to reduce mortality by allowing diagnosis before disease dissemination. Due to earlier diagnosis, screen-detected breast cancers (SDBCs) are smaller and less commonly spread to the axillary lymph nodes, which is partly an advantage gained by screening and lead time [1–3]. Biologically, SDBCs are often well-differentiated, hormone receptor-positive, HER2 negative, and associated with low tumor proliferation activity (low Ki-67 expression) [1–5]. Because of the slower growth rate, these cancers are more likely detected by screening than rapidly growing cancers, which is called length bias [6].

Within the biological spectrum of breast cancers, those displaying gene amplification of human epidermal growth factor receptor 2 (HER2 +) and those that are triple-negative (TN, i.e., negative for ER, PR and HER2) are generally considered biologically aggressive tumor subtypes. For HER2 + breast cancer, the prognosis has improved due to the introduction of targeted therapies [7–9]. For TN breast cancer, no targeted oncological treatments are currently available. TN patients are treated with surgery, radiation, and chemotherapy [10].

Several studies have reported a lower proportion of HER2 + cancers in screen-detected than in clinically detected patients (age group 50 to 70 years). The proportion of HER2 + breast cancer in screen-detected tumors has varied between 8 and 18%, whereas in interval cancers, it has ranged from 13–44% [2, 3, 11–13]. In women who had not participated in screening, the proportion of HER2 + breast cancers ranged from 13%-28% in three studies [3, 13, 14]. A recent study from Ireland showed that in women aged 50–66 years, HER2 + cancers constituted 13% of those found in screening, 19% in interval cancers, and 18% in patients who had not participated in screening [15]. Similar patterns have also been found for TN cancers. Only 4–7% of screen-detected cancers are of the TN type, while 9–16% of interval cancers are of the TN type [2, 11, 12, 15]. Few earlier studies have compared clinical and biological features within the HER2 + and TN subtypes according to the method of detection. Dawson et al. reported slightly better overall survival among screen-detected HER2 + and TN cancer patients compared to interval cancer patients, but the differences were not statistically significant [2].

We aimed to explore the prognosis of HER2 + or TN breast cancers found by screening and by symptoms between two screening rounds and in women of screening age (50–69 years) who did not participate in screening.

## Materials and methods

### Setting

The target population for the mammography screening program was women aged 50–60 years in 2006–2007, women aged 50–64 in 2008–2009, women aged 50–66 in 2010–2011, and women aged 50–68 years since 2012. Women were invited for mammography screening every 2 years. Mediolateral, oblique, and craniocaudal views were available for both breasts. All mammograms were independently read by two radiologists. Switching from film mammography to full-field digital mammography (Senographe Essential and Senographe DS, GE Healthcare) took place in autumn 2007, and one more full-field digital mammography device was obtained in 2014 (MicroDose Mammography, Philips). The screening program complies with the European Guidelines for Quality Assurance in Mammographic Screening [16]. All women invited for screening were residents of the city of Tampere. During the study period, 12,131 women on average were invited yearly to the screening, and the participation proportion was 81.8% in the first screening round and 84.1% in subsequent rounds.

After double reading, suspicious and/or unclear mammograms were reviewed together, and a recall for additional examinations was decided by consensus. The recall rate was 2.9%. If the finding could not be proven benign, a biopsy was taken by core needle or vacuum-assisted needle biopsy together with a fine needle or core needle biopsy of suspicious nodes. The detection rate of malignant findings was 0.78%. Preoperative MRI was used in a minority of cases upon consideration of a multidisciplinary meeting.

Invasive breast cancers were treated by breast-conserving surgery or mastectomy. Sentinel lymph node biopsy was performed during surgery in 75% of screen-detected cancers. The frequency of sentinel node biopsies in operations of other cancers is not available. An axillary evacuation was performed for patients with sentinel node metastasis or preoperative evidence of axillary metastases. Postoperatively, the women received radiation therapy, adjuvant chemotherapy, and hormonal therapy according to the national guidelines. Trastuzumab has been included as part of adjuvant chemotherapy since 2006 for patients with HER2 + breast cancer. Neoadjuvant chemotherapy was given if the cancer was considered inoperable at the time of diagnosis.

### Study population

We included all women in the target population of the mammography screening program diagnosed with invasive breast cancer for the first time. Women with in situ cancers were excluded. Additionally, women with previous in situ or invasive breast cancer diagnoses in the same or contralateral breast were excluded.

A total of 823 breast cancers in 805 patients matching these criteria were identified from the local pathology database. Of these cancers, 572 (from 559 patients) were detected by screening (SDBC). The median age of patients with screen-detected cancer was 60. The number of cancers detected within two years after a normal mammography screening was 170 (interval breast cancers, IBCs) in 168 patients, with a median age of 59. Altogether 742 cancers were detected in 727 women who did participate in screening (participation in screening breast cancers = PSBCs), while in women who did not participate in the screening program at all or skipped at least the previous screening mammography (nonparticipants), 60 patients had 63 breast cancers. For 18 women (2.2%) with invasive cancer, no information about screening could be found. These cases were included in the nonparticipants (no participation in screening breast cancers = NSBCs) group. The median age for these women was 59.

### Data

The clinical, histopathological, biomarker, and follow-up data were collected retrospectively from the medical records and mammography screening database. Neoadjuvant chemotherapy was given in 10 SDBC, 8 IBC, and 23 NSBC cases, and in those cases, tumor size refers to the largest tumor diameter measured in mammography or ultrasound. Patients with metastases detected within two months from diagnosis were regarded as metastatic at entry and were therefore not included in the analysis of distant disease-free survival. All HER2 oncogene diagnoses were based on chromogenic in situ hybridization and analyzed in a single laboratory.

### Statistical analyses

Frequency tables were analyzed by two-tailed Fisher’s exact test. The date of the last follow-up for relapse-free or living patients was 2-12-2019. The median follow-up was 8 years when the time to distant metastasis, death, or loss from follow-up was recorded as end-points. Distant-metastasis-free survival time was estimated using the Kaplan–Meier method log-rank test used for comparisons between the groups. A Cox proportional hazards model was used for multivariable analyses in Stata. Analyses comparing biologic subgroups by the method of detection were performed by adding an interaction term to a model with the main effects and assessing the improvement in fit with a likelihood ratio test. The proportionality assumption was evaluated based on Schoenfeld residuals. Analyses were truncated at eight years, as proportionality was not confirmed in the entire follow-up. The results were, however, qualitatively similar for both full and truncated follow-up.

## Results

### General histopathological features

The majority of SDBCs were smaller than 2 cm pT1 (82%) and node-negative pN0 (69%), while the proportions were smaller for IBCs and NSBCs (pT1 53%, pN0 52% and pT1 43%, pN0 46%, respectively, *p* < 0.0001 for both comparisons to SDBCs). Biologically, IBC and NSBC were more aggressive than SDBC. Histological grade 3 was found only in 15% of SDBC, but in 35% of IBC, in 31% of NSBC (both *p* < 0.0001), which demonstrates de-differentiation during tumor growth. A high tumor proliferation rate (Ki-67 ≥ 15%) was found in 35% of SDBC, while 48% of IBC and 51% of NSBC (both *p* < 0.01). The proportion of HER2-positive tumors was 10% in SDBC, 15% in IBC and 16% in NSBC (*p* = 0.051, *p* = 0.07, respectively). In SDBC the proportion of TN cases were 5%, in IBC 6%, and in NSBC 11% (*p* = 0.31, *p* = 0.03, respectively) (Table 1).

**Table 1:** Histopathological features of invasive breast cancers according to the method of detection

	Screen-detected cancers (SDBC)	Interval cancers (IBC)	No participation in screening cancers (NSBC)	*p*-value
Number of tumors	572	170	81	
Tumor size				
Smaller than 2 cm	469 (82%)	90 (53%)	35 (43%)	< 0.0001 (SDBC versus IBC), < 0.0001 (SDBC versus NSBC), < 0,0001 (PSBC versus NSBC)
2 cm or larger	101 (18%)	78 (46%)	45 (56%)	
Tumor size unknown	2 (< 1%)	2 (1%)	1 (1%)	
Nodal status				
pN0	396 (69%)	88 (52%)	37 (46%)	< 0.0001, < 0.0001, < 0,001
pN +	176 (31%)	82 (48%)	44 (54%)	
Distant metastasis				
M0	568 (99%)	165 (97%)	63 (78%)	0.0335, < 0.0001, < 0.0001
M +	4 (1%)	5 (3%)	18 (22%)	
Histological grade				
Grade 1	211 (37%)	27 (16%)	17 (21%)	
Grade 2	265 (46%)	76 (45%)	27 (33%)	
Grade 3	88 (15%)	59 (35%)	25 (31%)	< 0.0001, < 0.0001, 0.0034*
Grade unknown	11 (2%)	8 (5%)	12 (15%)	
Ki-67 < 15%	367 (64%)	89 (52%)	40 (49%)	0.0052, 0.0099, 0.0413
Ki-67 ≥ 15%	202 (35%)	81 (48%)	41 (51%)	
Ki-67 unknown	3 (< 1%)	0	0	
Luminal type (ER + and/or PR + , HER2-)	485 (85%)	133 (78%)	59 (73%)	
HER2 +	57 (10%)	26 (15%)	13 (16%)	0.0509, 0.0737, 0.1339**
Triple negative	27 (5%)	11 (6%)	9 (11%)	0.3120, 0.0267, 0.0328***
Unknown receptor status	3 (< 1%)	0	0	

Fisher’s exact test was used for *p-*value calculation

*p-*values for the SDBC versus IBC, SDBC versus NSBC, and PSBC (SDBC + IBC) versus NSBC groups

*pN0,* no postoperative axillary metastases; *M0,* no distant metastases; *pN* + axillar and *M* + distant metastases; *Ki-67,* tumor proliferation rate; *ER,* estrogen receptor; *PR,* progesterone receptor; *HER2,* HER2 oncogene amplification*; triple-negative,* negative for ER, PR, and HER2

*p-value refers to Grade 3 vs. Grade 1 + Grade 2, **p-value refers to HER2 + vs Luminal type, ***p-value refers to TN vs Luminal type

In the study population of 806 patients with 824 breast cancers, 96 (12%) cancers were HER2 + and 47 (6%) were TN. Even though their proportion in HER2 + cases was lower in SDBC than in the other groups, more than half of the HER2 + breast cancers, as well as the TN breast cancers were found by screening (59% and 57%, respectively). A quarter of the cases in the more aggressive subgroups were interval cancers (27% and 23%, respectively). Digital screening (31 of 37) mammography views of interval HER2 + and TN patients were reanalyzed. Only five cases had minimal changes in the place where cancer later appeared. Thus, almost all of them were true biological intervals and not false-negative interpretations of screening mammograms.

### Biological and clinical features of HER2 + and TN cancers

Of the HER2 + and TN breast cancers detected by screening, the majority were smaller than 2 cm (79% and 81%, respectively) and were node-negative (pN0 84% and 70%, respectively). In contrast, of the HER2 + interval cancers diagnosed between the two screening rounds, 58% were 2 cm or larger, and 62% had metastases in the axilla, while HER2 + breast cancers detected in screening nonparticipants were mostly larger than 2 cm (77%), node-positive (92%) and 62% of them had distant metastasis already at the time of diagnosis (Table 2). The majority of interval TN breast cancers were smaller than 2 cm (73%), but almost all TN breast cancers (89%) that were diagnosed in women who did not participate in screening were 2 cm or larger (Table 3). The median tumor size was 13 mm for all luminal types, 16.5 mm for HER2 + , and 14.5 mm for TN tumors. The Ki-67 index medians were 10, 33, and 46, respectively. Regardless of the method of detection, HER2 + and TN carcinomas were predominantly of histological grade 3 (in screening HER2 + 53% and TN 70%, interval 77% and 100%, no participation 62% and 78%) and displayed a high proliferation rate (Ki-67) (Tables 2, 3).

**Table 2:** Histopathological features of HER2 + breast cancers in the detection groups

	Screen-detected cancers (SDBC)	Interval cancers (IBC)	No participation in screening cancers (NSBC)	*p*-value
Number of tumors	57	26	13	
Tumor size				
Smaller than 2 cm	45 (79%)	11 (42%)	2 (15%)	0.002 (SDBC versus IBC), < 0.0001 (SDBC versus NSBC), 0.0011 (PSBC versus NSBC)
2 cm or larger	12 (21%)	15 (58%)	10 (77%)	
Size unknown	0	0	1 (8%)	
Nodal status				
pN0	48 (84%)	10 (38%)	1 (8%)	< 0.0001, < 0.0001, < 0.0001
pN +	9 (16%)	16 (62%)	12 (92%)	
Distant metastasis				
M0	57 (100%)	26 (100%)	5 (38%)	1.00, < 0.0001, < 0.0001
M +	0	0	8 (62%)	
Histological grade				
Grade 1	2 (4%)	0	0	
Grade 2	19 (33%)	6 (23%)	1 (8%)	
Grade 3	30 (53%)	20 (77%)	8 (62%)	0.1364, 0.1353, 0.2605 *
Grade unknown	5 (9%)	0	4 (31%)	
ER + and/or PR +	34 (60%)	10 (38%)	8 (62%)	0.0978, 1.000, 0.7760
ER-,PR-	23 (40%)	16 (62%)	5 (38%)	
Ki-67 < 15%	7 (12%)	6 (23%)	0	0.3508, 0.3361, 0.2031
Ki-67 ≥ 15%	49 (86%)	20 (77%)	13 (100%)	
Ki-67 unknown	1 (2%)	0	0	

Fisher’s exact test was used for *p-*value calculation

*p-*values compare SDBC versus IBC and SDBC versus NSBC and PSBC (SDBC + IBC) versus NSBC groups

*pN0,* no postoperative axillary metastases; *M0,* no distant metastases; *pN* + *,* axillar and *M* + *,* distant metastases; *Ki-67,* tumor proliferation rate; *ER,* estrogen receptor; *PR,* progesterone receptor; *HER2,* HER2 oncogene amplification

*p-value compares Grade 3/(Grade 1 + Grade 2)

**Table 3:** Histopathological features of TN breast cancers in the detection groups

	Screen-detected cancers (SDBC)	Interval cancers (IBC)	No participation in screening cancers (NSBC)	*p*-value
Number of tumors	27	11	9	
Tumor size				
Smaller than 2 cm	22 (81%)	8 (73%)	1 (11%)	0.4026 (SDBC versus IBC), 0.0002 (SDBC versus NSBC), 0.0002 (PSBC versus NSBC)
2 cm or larger	4 (15%)	3 (27%)	8 (89%)	
Size unknown	1 (4%)	0	0	
Nodal status				
pN0	19 (70%)	6 (55%)	3 (33%)	0.4573, 0.1111, 0.1292
pN +	8 (30%)	5 (45%)	6 (67%)	
Distant metastasis				
M0	26 (96%)	10 (91%)	7 (78%)	0.5, 0.1479, 0.1605
M +	1 (4%)	1 (9%)	2 (22%)	
Histological Grade				
Grade 1	1 (4%)	0	0	
Grade 2	5 (19%)	0	0	
Grade 3	19 (70%)	11 (100%)	7 (78%)	0.1479, 0.2964, 0.5671*
Grade unknown	2 (7%)	0	2 (22%)	
Ki-67 < 15%	5 (19%)	0	1 (11%)	0.2949, 1.000, 1.000
Ki-67 ≥ 15%	22 (81%)	11 (100%)	8 (89%)	

Fisher’s exact test was used for *p-*value calculation

*p-*values compare SDBC versus IBC and SDBC versus NSBC and PSBC (SDBC + IBC) versus NSBC groups

*pN0,* no postoperative axillary metastases; *M0,* no distant metastases; *pN* + axillar and *M* + distant metastases; *Ki-67* tumor proliferation rate; *TN,* triple-negative, negative for *ER* estrogen receptor; *PR,* progesterone receptor; and *HER2,* HER2 oncogene amplification

*p-value compares Grade 3/(Grade 1 + Grade 2)

### Prognosis

**Fig. 1:**
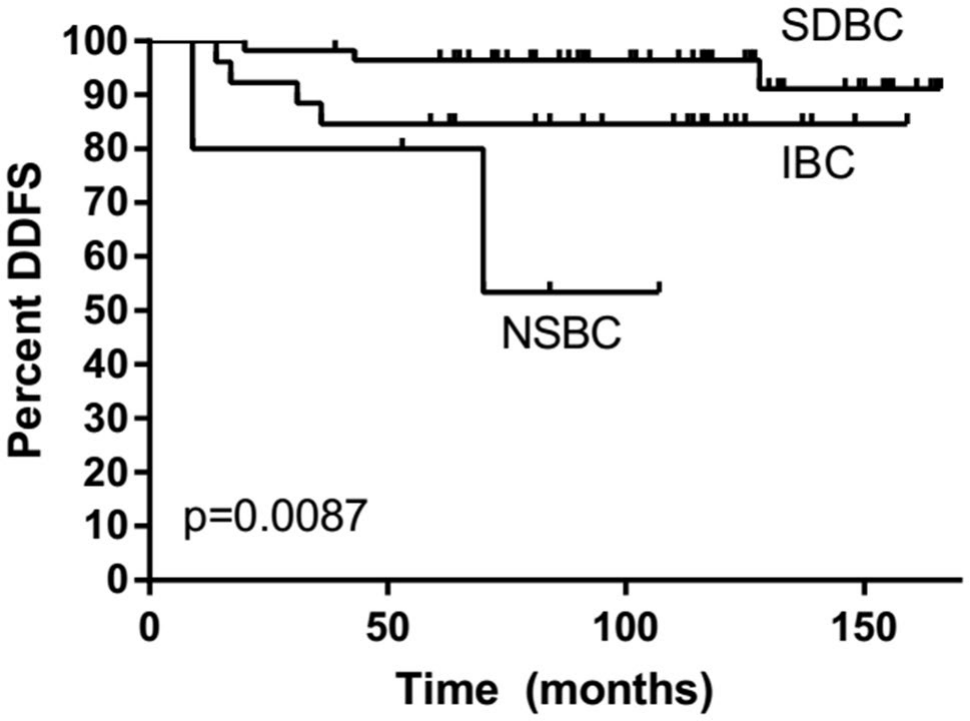
Distant disease-free survival (DDFS) of HER2-positive (estrogen and progesterone receptor-negative or positive and HER2 oncogene positive) breast cancers among screen-detected (SDBC), interval (IBC), and nonparticipant (NSBC) cases

**Fig. 2:**
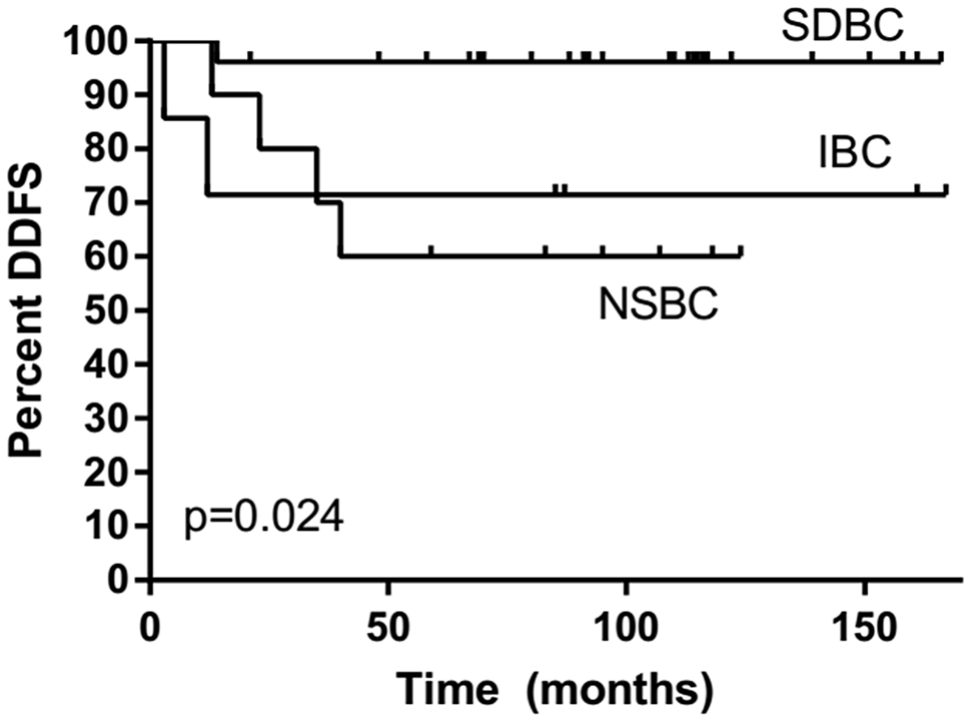
Distant disease-free survival (DDFS) of triple-negative (estrogen and progesterone receptor-negative and HER2 oncogene negative) breast cancers among screen-detected (SDBC), interval (IBC), and nonparticipant (NSBC) cases

**Fig. 3:**
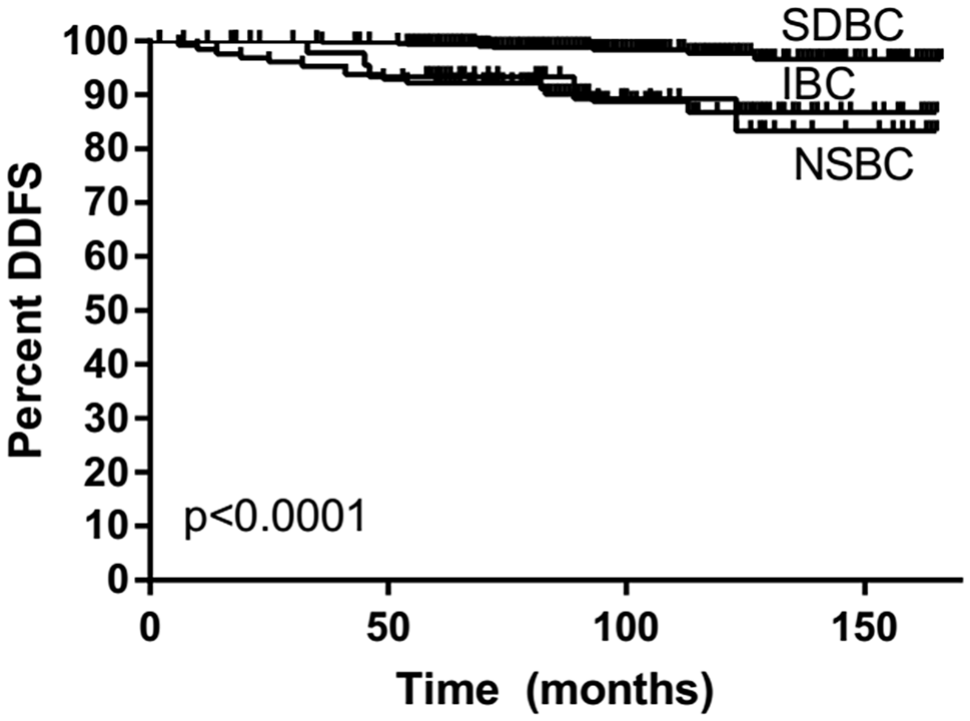
Distant disease-free survival (DDFS) of luminal type (estrogen and/or progesterone receptor-positive and HER2 oncogene negative) breast cancers among screen-detected (SDBC), interval (IBC), and nonparticipant (NSBC) cases

Only 1% of SDBCs, 3% of IBC, and 22% of NSBC had distant metastases (M +) at the time of diagnosis (Table1). HER2 + breast cancers found in women who had participated in screening (SDBC and IBC) were all distant metastasis-free at the time of diagnosis (Table 2). During the 8-year median follow-up, distant metastasis appeared in 44 (6%) of all patients who did not have distant metastasis (M0) at the time of diagnosis. In SDBC, distant metastases appeared in only 5% of HER2 + cancer patients, 4% of TN cancer patients, and 1% of patients with luminal tumor type, while in IBC, the corresponding figures were 15, 40, and 12 and NSBC 40, 29, and 12. The distant disease-free survival of screen-detected HER2 + and TN cancers was better than that of interval and nonparticipant cancers (age-adjusted HR = 0.16, 95% CI 0.03–0.81 and HR = 0.09, 95% CI 0.01–0.79, respectively). It should also be noticed that the distant disease-free survival of nonparticipant HER2 + and TN cancers was worse than that of PSBCs (age-adjusted HR = 2.52, 95% CI 0.63–10.11 and HR = 5.30, 95% 1.16–24.29, respectively). Distant disease-free survival by the method of detection among HER2 + (Fig. 1), TN (Fig. 2), and luminal type (Fig. 3) cases is illustrated in Kaplan–Meier plots. SDBC had the most favorable prognosis in all three cancer subgroups, with no major differences by the method of detection across the tumor types (interaction *p* = 0.79 in Cox analysis truncated at eight years, median follow-up then 92 months). Of the screen-detected cases, the patients with luminal-type cancer tended to experience superior distant metastasis-free survival. There were also indications for prognosis being worst for patients with TN SDBC (HR = 3.78, 95% CI 0.44–32.37, compared to luminal-type SDBC), though the difference from HER2 + cancer was not significant (HR = 3.27, 95% CI 0.63–16.87, for HER + relative to luminal-type cases). After adjustment of the Ki-67 index the differences disappeared (TN 1.14, 95% CI 0.36–3.66, HER2 0.97, 95% CI 0.32–2.98).

## Discussion

In Finland, the proportion of HER2 + breast cancers in all age groups has been approximately 15% [17]. Among patients aged 50 to 69 years in our study, the proportion of HER2 + breast cancers was 12%, and in screening, the proportion of HER2 + cancers was only 10%. The lower proportion is partly due to the higher percentage of HER2 + and TN cancers in patients younger than 50 years. The larger proportion among IBC (15%) can be explained by the fact that HER2 + cancers are rapidly growing, with a shorter asymptomatic and preclinical detectable phase, and therefore often surface clinically between screening rounds. The lower frequency of screen-detected invasive HER2 + cancers might be explained by the increased presentation of screen-detected in situ cancers, which are often HER2 + . Digital mammography detects more in situ cancers than film mammography [18], and currently, when older women are invited to the screening, part of their cancers are detected as in situ cancer too. In our study, the proportion of TN breast cancers in patients aged 50–69 years was 6%, and the proportion of SDBCs was 5%. Previous studies have shown similar results, with 4–7% of TN cases in screen-detected breast cancers among women aged from 50 to 65/70 years [2, 11, 12, 15]. In our study in 50–69-year-old women the proportion of TN cancers among NSBCs was 11% while of PSBCs it was 5%. This can be due to the fact that part of the hormone-positive cancers are so slow-growing that they don´t become symptomatic until after screening age, but also some hormone-positive tumors at an early stage might change to hormone-negative cancers if left to a late-stage but it is not known how much this is happening.

Even though HER2 + and TN breast cancers are considered rapidly growing, the majority (59% and 57%, respectively) of them were detected by population-based screening in this age group. This is in line with a previous study [19]. In our study, SDBCs were 77% of the PSBCs. The proportions were almost the same in HER2 + (69%) and TN (71%) groups which suggest only a minor length bias phenomenon. This shows that HER2 + and TN cancers can be detected by screening almost as well as cancers in general.

Tumor size, nodal status, and distant metastases are the main determinants of patient prognosis. Screen-detected cancers are typically small and node-negative. In our study, screen-detected cancers were smaller than 2 cm and node-negative in HER2 + (79%, 84%) and TN (81%, 70%) cases detected by screening. However, in early pT1abN0 tumors, HER2 + cases may have a substantial risk for recurrence unless treated with adjuvant therapy [20, 21]. For this reason, adjuvant therapy is also recommended for patients with small node-negative HER2 + breast cancer [20, 22]. TN cancers have a substantial risk of relapse, irrespective of additional variables such as grade, lymph node status, and tumor size [23].

HER2 + and TN cancers are considered to be aggressive and in our study, despite the detection method HER2 + and TN breast cancers were often histological grade 3 and even more often displayed a high proliferation rate (Ki-67). It is also known that they have a poorer prognosis than other molecular types of breast cancers. Results from the Swedish Two-County Trial have shown that mortality from histological grade 3 breast cancers can be decreased by invitation to screening [24]. And the 20-year follow-up showed that participation in screening degreased fatal breast cancer incidence significantly compared to those who did not participate[25]. We chose to invest women who were all invited to screening and among them, the best distant disease-free survival of HER2 + and TN cancers was in screen-detected cancers. Also, the prognosis of these cancers detected in women who participated in screening was far better than those who did not. Differences in disease-free survival between screen-detected luminal and aggressive cancers (HER2 + and TN) disappeared after adjustment for the Ki-67 tumor proliferation marker, which suggests that tumor aggressiveness is mediated by more rapid cell turnover reflected by Ki-67.

Among HER2 + cancers, the survival difference seems to be explained by an earlier stage of cancer, but in TN cancers, many interval cancers were small, while almost one-third of screen-detected TN cancers were already node-positive. In TN cancers, tumor size was only weakly correlated with lymph node metastasis [23], but our findings showed that screening can detect TN cancers early enough to influence prognosis. Some studies have reported better survival in screen-detected than clinically detected HER2 + and TN cancers [2, 26–28], but we found no published reports of more favorable survival in screen-detected than interval HER2 + and TN cancers.

A strength of our study is the population-based approach, which contains almost complete information for every single case and contains comprehensively and systematically defined molecular subtypes. Undefined cases are due to lack of data because of the minimal proportion of invasion in some cases of ductal in situ cancer.

The limitations of our study are that the evaluation is based on the results of a single screening unit and thus may not be readily generalizable to other settings. Additionally, the number of cases is limited.

## Conclusion

Although screening mainly detects slowly growing, hormone receptor-positive and HER2-negative cancers, our findings also indicate that a substantial proportion of HER2 + and TN breast cancers can be detected by screening. The screen-detected HER2 + and TN cancers were diagnosed at an earlier stage, and their prognosis was far better than those detected by the symptoms and the prognosis was worse in women who did not participate in screening compared to women who did.

## Abbreviations

HER2 +: HER2 oncogene positive
TN: Triple-negative
ER: Estrogen receptor
PR: Progesterone receptor
HOR: Estrogen and progesterone receptor
SDBC: Screen-detected breast cancer
IBC: Interval breast cancer
PSBC: Participation in screening breast cancer
NSBC: No participation in screening breast cancer
